# Differences in Cost-Effectiveness of Adherence to Nutritional Recommendations: Why, Where, and What?

**DOI:** 10.3390/ijerph20010772

**Published:** 2022-12-31

**Authors:** Aline Veroneze de Mello, Flavia Mori Sarti, Marilisa Berti de Azevedo Barros, Moises Goldbaum, Chester Luiz Galvão Cesar, Regina Mara Fisberg

**Affiliations:** 1 School of Public Health, University of Sao Paulo, Sao Paulo 01246-904, Brazil; 2 School of Medical Sciences, State University of Campinas, Campinas 13083-894, Brazil; 3 School of Medicine, University of Sao Paulo, Sao Paulo 01246-903, Brazil

**Keywords:** cost-effectiveness, street markets, diet quality, nutritional recommendations, income

## Abstract

Cost-effectiveness analysis of diets may comprise an important tool to promote food security; however, studies show divergent evidence regarding the relationship between diet quality and cost in diverse populations. Thus, this study assesses differences in cost-effectiveness ratios regarding adherence to nutritional recommendations using data representative of the population level in Sao Paulo municipality, Brazil. Information from adolescents and adult individuals (*n* = 1742) was used to estimate diet quality and cost in 2015. Differences in cost-effectiveness ratios were investigated through application of two diet quality indexes and exploration of individuals’ personal and contextual characteristics. Results indicated that higher diet cost was associated with higher adherence to nutritional recommendations at the national level and inversely associated with adherence to international recommendations. Purchasing foods in street markets was linked to healthier diets at lower costs, and protein consumption was associated with higher diet cost regardless of diet quality; however, diet quality was linked to type of protein consumed by individuals. Differences in cost-effectiveness ratios were attributable to methodological choices in measuring dietary quality (why); individuals’ personal and contextual characteristics, in particular, access to retail equipment (where); and certain food choices (what). Therefore, cost-effectiveness analyses should be tailored to policy goals and local environments to ensure proper assessment of nutrition programs and to foster improvements in nutritional diet quality at lower cost.

## 1. Introduction

Recent evidence indicates significant association between nutritional quality of diets and reduction of relative risk for occurrence of chronic noncommunicable diseases (NCDs), including diabetes, heart disease, stroke, and cancer [1,2,3]. Initiatives for disease prevention and health promotion comprise main strategies in primary health care to reduce the occurrence of NCDs in different countries today. Thus, there are various proposals of nutritional recommendations and definitions of healthy diets aimed at guidance for adherence to favorable lifestyles [4,5].

Indices for the assessment of diet quality have been useful measures to investigate the role of nutritional recommendations in health promotion, providing information on food consumption patterns of populations [4]. At the international level, the World Health Organization proposes general nutritional guidelines in the report, “Healthy Eating”, indicating that diets with higher nutritional quality should include at least 400 g of fruits, and vegetables per day; less than 5% of total energy intake from sugars; less than 10% of total energy intake from saturated fats; and less than 2000 mg of sodium per day. Other indices of nutritional quality of diets synthesize diverse dimensions of interest in individuals’ eating patterns being adopted for monitoring populational adherence to healthy lifestyles and enabling the assessment of potential food substitutions that improve the nutritional quality score and/or optimize the diet cost [4,6,7,8].

The adoption of cost-effectiveness analysis of diets may support promotion of food security through the comparison of costs and outcomes related to diverse food consumption patterns, providing information for evidence-based decision-making in public policies. Nevertheless, studies show divergent evidence regarding the relationship between diet quality and costs in diverse populations. Studies conducted in developed countries point to higher dietary costs among individuals with higher adherence to nutritional recommendations and/or food guidelines compared to individuals with lower adherence [9,10,11,12]. Other studies in developing countries, such as Brazil, show contradictory evidence regarding the relation between diet quality and costs [8,13,14]. Yet, there is lack of discussion on the potential causes of differences identified in cost-effectiveness ratios of diet patterns investigated worldwide.

Food prices represent important barriers to changes in dietary patterns toward healthy diets, especially among middle- and low-income individuals and population groups in developing countries marked by considerable socioeconomic disparities [15,16,17,18]. In addition, the income level represents one of the main factors influencing diet quality in Brazil [19], accounting for a major part of inequality observed in the nutritional quality of diets consumed by individuals in the municipality of Sao Paulo between 2003 and 2015 [20].

Location of food purchases represents another important factor linked to diet quality, considering the various types of food retail with substantial differences in prices and assortment of products in Brazil [21,22]. Recent evidence showed that individuals prefer buying foods at supermarkets instead of street markets in Brazil due to convenience of purchasing assorted products in one single retail store. However, purchasing at supermarkets usually discourages acquisition of fresh foods, e.g., fruits and vegetables. Conversely, street markets with an itinerant location are supported by the government in Brazil, being particularly important for providing access to fresh food in Brazilian municipalities due to the wide variety and low prices of fresh foods supplied [21,22].

Considering the potential role of evidence on cost-effectiveness for guiding public policy, it is important to investigate potential causes of differences in diet quality and costs assessed under diverse conditions, particularly in developing countries. Thus, the present study assesses differences in cost-effectiveness of adherence to nutritional recommendations in Sao Paulo municipality, Brazil, using two diet quality indexes, according to individuals’ personal and contextual characteristics.

## 2. Materials and Methods

### 2.1. Study Sample

The Sao Paulo Health Survey (ISA-Capital 2015), conducted in the municipality of Sao Paulo from September 2014 to December 2015, was based on interviews of individuals selected through a complex sampling process in two stages. The sample was selected based on geographical stratification of the population in five domains, corresponding to the Health Coordinating Areas of Sao Paulo: North, Midwest, Southeast, South, and East. In the first sampling stage, thirty urban census tracts were randomly selected from each geographic area, totaling 150 primary sampling units. In the second sampling stage, approximately eighteen households were systematically selected in each census tract, considering six demographic domains of sample planning referring to age/sex of individuals (male adolescents aged 12–19 years, female adolescents aged 12–19 years, male adults aged 20–59 years, female adults aged 20–59 years, male older adults aged ≥ 60 years, and female older adults aged ≥ 60 years).

The minimum sample size was 300 individuals per age/sex domain, which allowed for estimates of proportions of 0.5 with a sampling error of 7%, considering 95% confidence level and effect size of 1.5 [23]. Individuals within selected households who met the criteria for inclusion in the sample were invited to participate in the survey. A total of 5942 households were included in the initial sample; however, 8.0% were vacant households, and 70.3% of households had residents providing information on sex and age groups of interest in the survey, resulting in 4177 households with 4059 eligible residents who agreed to participate in the survey (73.4% of the eligible residents). Among eligible residents, 1742 individuals (adolescents, adults, and older adults) were randomly selected for two additional stages of data collection regarding food intake during the previous 24 h, being included in the 2015 ISA-Nutrition subsample [24]. The present study was based on the subsample including adolescents (*n* = 554), adults (*n* = 643), and older adults (*n* = 545), i.e., data from 1742 individuals.

### 2.2. Data Collection

Survey data were collected using a semi structured questionnaire with 16 thematic sections (including socioeconomic, demographic, health, and lifestyle information) in an electronic format through tablets during a face-to-face interview with trained interviewers in individual meetings.

Dietary intake data were obtained during a face-to-face interview and another telephone interview on two nonconsecutive days, based on 24-h food recall (24HR) using the multiple pass method (MPM) [25]. Participants were instructed to report dietary intake, preparation method, ingredients, and brands of food items.

The analysis of nutritional composition of diets reported by the participants was estimated using the Nutrition Data System for Research (NDSR-2014) software (Nutrition Coordinating Center, University of Minnesota, Minneapolis, MN, USA). Interview methods are presented in a previous publication referring to the technical guide for ISA-Capital interview procedures [26].

### 2.3. Direct Cost of Diets

Data on food consumption collected in the survey were linked to information on food prices from the Household Budget Survey of the Brazilian Institute for Geography and Statistics (POF-IBGE), conducted from May 2008 to May 2009, through database linkage techniques due to the absence of food prices in the health survey. Databases comprising individual and household level information from POF-IBGE are publicly available on the official website of the institution [27].

POF-IBGE 2008–2009 was the most recent survey representative at the population level to include food prices during the implementation of the present study. Thus, prices of food items in Sao Paulo municipality from POF-IBGE 2008–2009 were updated to 2015 using their corresponding inflation rates to allow identification of relative prices in 2015.

The database linkage process was performed using a set of criteria to match prices of food items from the POF-IBGE with food items consumed by individuals interviewed in the ISA-Nutrition; geographic (Sao Paulo municipality), economic, and demographic characteristics (household income per capita and age of residents), details were previously described in another publication [28].

Correction factors and cooking indices were applied to each food reported in the 24HR to obtain net weight and estimate prices per gram of food ready for consumption, considering that prices were available per unit purchased (in grams or milliliters) [29,30].

Finally, the direct diet cost per calorie was calculated by multiplying prices per gram and amounts of food items consumed. Diet costs per calorie were updated and converted into International Monetary units (IM) in 2019, using Consumer Prices Index and Purchase Power Parity conversion factors referring to Brazil, available at the website of the World Bank [31].

### 2.4. A priori Dietary Pattern (Brazilian Health Eating Index-Revised (BHEI-R))

The nutritional quality of the diet was assessed through the BHEI-R, which is an indicator based on the Healthy Eating Index, adapted and validated for the Brazilian population. The index is based on scores attributable to foods, nutrients, and other dietary components based on nutritional recommendations and/or associated health outcomes, including twelve components (nine food groups, two nutrients, and the sum of energy from saturated fat, alcohol, and added sugar-SoFAAS).

The consumption of fruits, whole fruits, dark green and orange vegetables and legumes, and total cereals and whole grains may be scored between 0 and 5 points. The consumption of milk and dairy products, meats, eggs and legumes, oils (vegetable oils, seed oils, and fish oils), saturated fat, and sodium may be scored between 0 and 10 points; and the intake of saturated fat, alcohol, and added sugar may be scored between 0 and 20 points. Scores attributable to saturated fat, sodium, and SoFAAS present an inverse relation to nutrient intake, i.e., consumption exceeding the recommendations receives lower scores, and consumption within recommended limits receives higher scores. The BHEI-R corresponds to the sum of scores for the twelve aforementioned components, ranging from 0 to 100 points, with 0 being attributable to diets with the lowest nutritional quality and 100 attributable to diets with the highest nutritional quality. The analyses performed in the present study considered categorization of the BHEI-R into quintiles, with the first quintile representing the worst diet quality and the fifth quintile representing the best diet quality.

### 2.5. International Recommendations of the World Health Organization (WHO)

Considering the general nutritional guidelines proposed by the WHO on the report “Healthy Eating”, we estimated diet quality using the international recommendations to allow comparison of cost-effectiveness ratios obtained using the BHEI-R and the WHO indicator. Total energy was calculated by applying the conversion factors proposed by FAO/WHO (2003) for carbohydrates (4 kcal/g), protein (4 kcal/g), fat (9 kcal/g), alcohol (7 kcal/g), and total fiber (2 kcal/g). Adjustment of the energy intake distribution based on two 24HRs was estimated through the Multiple Source Method (MSM), a statistical method aimed at adjusting intrapersonal dietary variability to estimate individuals’ usual intake.

### 2.6. Food Groups

The categorization of food items into food groups was performed to allow identification of main food groups and their food items contributing to diet quality and costs. Food items consumed by individuals were grouped using the “What we eat in America?” Food Classification System (WWEIA), based on the National Health and Nutrition Examination Survey (NHANES) database, adapted to the Latin American context. Details on the adaptation are available in another publication [32].

### 2.7. Additional Variables

Considering potential sources of confounding effects, the following variables were included in the statistical analyses: sex (male or female), age (continuous), household per capita income (continuous and categorized in ≤1 and >1 Brazilian minimum wage per capita, with the minimum wage being equivalent to BRL 788 in 2015, or IM 457.61 in 2019) [31,33], social class (considering ownership of assets and educational level, categorized into: A, B1 + B2, C1 + C2, D + E) [34], skin color (white and others), location of food purchase (minimarket, supermarket, whole foods market, and wholesaler), frequency of food purchases in street markets (categorized into: none, 1 time per month, 1 to 2 times per month, 1 to 2 times per week, and 3 or more times per week). Questions without response were coded missing.

### 2.8. Statistical Analysis

The analyses were conducted using the statistical software Stata, version 13.1, adopting the survey mode to ensure population representativeness. Descriptive analyses were performed using means, standard error, and 95% confidence intervals (95%CI).

The proportion of food costs in relation to household income per capita was based on the comparison between mean diet cost and household income per capita per day. We applied a linear trend test (Stata nptrend command) to identify associations between food costs and diet quality (BHEI-R) or adherence to WHO recommendations, considering the descriptive level of *p* < 0.05.

Multiple linear regression models were estimated, adjusting for potential confounding effects (diet quality-BHEI-R, grams consumed, and energy, using the residual adjustment method), including sex, age, skin color, income, social class, location of food purchases, and frequency of buying at street markets. Variables were selected using *p* < 0.20 in the univariate model, followed by adoption of *p* < 0.05 for inclusion in the multivariate model. Residual analysis was performed to check for homoscedasticity of the errors in the regression, and we were able to verify absence of bias in the analysis.

The contribution of main food groups and food items in the diet cost, according to quintiles of diet quality score (1st. quintile—low-quality diet; 3rd. quintile—intermediate-quality diet; and 5th. quintile—high-quality diet), using the food classification system of the study “What We Eat In America food Classification System (WWEIA)” adapted for Latin America [32].

### 2.9. Cost-Effectiveness Analysis

The associations between direct cost and nutritional quality of the diet (BHEI-R score) was evaluated through cost-effectiveness analysis by estimating cost-effectiveness ratios (CER) and incremental cost-effectiveness ratios (ICER) [35], comparing individuals categorized into BHEI-R score quintiles.

The CER is estimated through the division of total costs incurred by individuals into each category by their corresponding health outcomes (Equation (1)):

(1) $$CER_{i}=\frac{C_{i}}{O_{i}}$$

where *CER_i_* = cost-effectiveness ratio of alternative *i*; *C_i_* = cost of alternative *i*; and *O_i_* = outcome (BHEI-R) associated with alternative *i*. The best alternative presents the lowest cost per unit of the diet quality score.

The ICER is calculated by comparing costs incurred by individuals in two categories and dividing by the corresponding difference in health outcomes (Equation (2)):

(2) $$ICER_{i,j}=\frac{C_{i}-C_{j}}{O_{i}-O_{j}}$$

where *ICER_i,j_* = incremental cost-effectiveness ratio of alternative *i* in relation to alternative *j*; *C_j_* = cost of alternative *j*; and *O_j_* = outcome associated with alternative *j*.

In the present study, the health outcome adopted was the nutritional quality of the diet, expressed through the BHEI-R score. Estimates of direct diet costs and health outcomes were based on the perspective of the payer (consumer), using short-term time horizon, referring to the period of food consumption recorded to avoid imposing unrealistic assumptions referring to final outcomes (onset of disease, death, or survival) deriving from healthy food consumption patterns. Thus, discount rates were not applicable in the analysis.

## 3. Results

Women, individuals with income higher than one minimum wage, individuals from social class A, and individuals declaring white skin color had higher diet costs compared to other individuals. Individuals who buy food mostly in whole foods stores and individuals with better diet quality also presented higher diet costs. In contrast, individuals who buy food at street markets presented lower diet costs, with lower diet costs generally being registered in relation to higher frequency of purchases at street markets (Table 1).

Individuals with income lower than minimum wage compromised major part of their daily per capita income on food (99.49%), unlike individuals with higher income (21.26%) and higher social class (A, 16.82%). White individuals and individuals with higher diet quality (5th quintile) also presented lower participation of food costs in the family budget (27.74% and 21.19%, respectively) (Table 1).

Moreover, individuals with higher income showed an increase of IM 0.52 in diet cost per BHEI-R point in comparison to individuals with lower income, while individuals from a higher social class (A) had an increase of IM 0.83 per BHEI-R point in comparison to individuals from a lower social class (D + E) (Table 1).

The highest direct diet cost was recorded among individuals in the higher BHEI-R quintile (75.0 BHEI-R points EP = 0.3; IM 11.12; 95% CI: 10.85; 11.39) and higher income (59.4 EP = 0.6; IM 11.23; 95% CI 10.98; 11.48), compared to individuals in the lower BHEI-R (40.2 EP = 0.4; IM 10.25; 95% CI 9.90; 10.60) and lower income (56.7 EP = 0.5; IM 9.86; 95% CI 9.67; 10.05). An increase of IM 0.03 in the diet cost was observed for each additional point in the BHEI-R, comparing individuals in the higher with lower diet quality quintiles (Table 1).

Individuals buying foods at street markets ≥ 1 time per week had lower diet costs (IM 9.91; 95% CI 9.80; 11.99 vs. IM 11.27 95% CI 9.90; 12.64), in addition to better diet quality (59.1 EP = 0.54 vs. 57.9 EP = 0.6), compared to individuals who do not buy at street markets (Table 1). Individuals buying foods at street markets ≥1 time per week had savings of IM 2.04 per BHEI-R point compared to individuals not shopping at street markets (Table 1).

Higher adherence to the nutritional recommendations represented by the BHEI-R (between the 1st and 5th quintile of diet quality) was associated with an increase in diet costs (nptrend: *p* < 0.001), except regarding the location of food purchases (whole foods market, minimarket, and wholesaler) (Table 2).

Contrarily to results obtained in the analysis of adherence to the Brazilian nutritional recommendations, higher adherence to WHO recommendations represented lower cost (IM 10.97; 95% CI 9.78; 11.41). That is, individuals who had a higher intake of fruits and vegetables (>400 g/day), lower intake of added sugar (<5% of total calories in the day), saturated fat (<10% of total calories in the day), and sodium (<2000 mg per day) presented lower dietary cost compared to individuals without adherence to the proposed recommendations (IM 12.03; 95% CI 11.38; 12.68) (Table 3). The adherence to WHO recommendations represented savings of IM 0.15 per BHEI-R point compared to individuals without adherence to the recommendations (Table 3).

Considering the contribution of specific food items in the diet costs, the group of meats represented a higher proportion of diet costs for all quintiles of diet quality (24.4% in the worst-quality diet; 29.5% in the intermediate-quality diet; 29.6% in the best-quality diet), followed by nonalcoholic beverages (16.1%; 13.7%; 12.93%, respectively) (Table 4).

Sweet and salty snacks represented the third group with highest contribution to diet costs among individuals with low quality diet (12.9%; IM 1.15; EP = 0.06), while the vegetable group represented the third highest cost for individuals with intermediate (10.7%; IM 0.96; EP = 0.05) and high-quality diets (12.93%; IM 1.12; EP = 0.08) (Table 4).

On the one hand, the food group with the lowest share of diet costs was oils and fats (<2% at any diet quality level). On the other hand, low-quality diets presented the group of fruits in the second lowest share of diet costs (5.24%; IM 0.47; EP = 0.04); while high-quality diets presented the group of sweet and salty snacks corresponding to the second lowest share of diet costs (6.7%; IM 0.57; EP = 0.04), indicating differences in choices of intermediate snacks among individuals with different levels of diet quality (Table 4).

Considering the main food items contributing to diet costs in each food group, diets with low nutritional quality (1st quintile) showed a higher share of costs due to red meat, coffee, breads, rice-based dishes, and potato chips. In contrast, higher quality diets (5th quintile) had greater participation in costs due to chicken, coffee, bread, skimmed milk, and potato, reflecting choices of foods with lower fat content compared to individuals with lower diet quality (Table 4).

## 4. Discussion

The present study represents one of the first investigations performed in Brazil of the association between diet quality and costs, based on the adherence to nutritional recommendations (a priori dietary pattern), using population-level data to present a cost-effectiveness analysis. In addition, we explored potential differences in the estimation of the cost-effectiveness of diets attributable to methodological changes referring to the choice of diet quality index (why) and effects of individuals’ personal and contextual characteristics, particularly referring to location of food purchases (where) and food choices (what) according to income and social class.

We identified that higher adherence to dietary recommendations usually represents greater diet costs. Furthermore, the results showed lower costs of diets that presented higher adherence to the WHO recommendations compared to diets with lower adherence, possibly due to low specificity of the WHO recommendations, which are based on four general parameters to allow adaptation for application in different countries. Therefore, the present study identified major changes in cost-effectiveness ratios according to two different measures of diet quality based on similar concepts of healthy eating, showing that international nutritional recommendations, e.g., WHO recommendations, may be generalized in relation to population-specific nutritional requirements and cultural background, which may be represented with higher accuracy by national nutritional guidelines, e.g., BHEI-R, which was adapted to suit food consumption patterns of the Brazilian population.

In addition, the adherence to WHO recommendations presents fewer criteria with a simple scoring system, which may be unable to capture slight changes in dietary patterns, while the BHEI-R encompasses twelve components with different weights, allowing us to identify minor changes in diet composition. In general, a major part of the differences in diet costs attributable to adherence to the WHO recommendations were driven by costs associated with the consumption of fruits and vegetables [36,37].

The relationship between diet quality and cost has been investigated in different countries, considering its implications to public health. A systematic review and meta-analysis on the costs of different dietary patterns identified studies conducted in the United States, Canada, Europe (Spain, France, Netherlands, and Sweden), South Africa, New Zealand, Japan, and Brazil, indicating that healthier dietary patterns present higher costs than unhealthier diets, based on the standardization of daily diets with 2000 kcal. The differences identified in diets costs in 2011, according to nutritional quality, was approximately IM 1.50 additional per person per day for adherence to healthier diets, equivalent to IM 1.70 in 2019 [21].

Additionally, the present study indicated that individuals with recommended consumption of fruits and vegetables had savings of IM 0.11 per point of BHEI-R compared to individuals without adherence to the recommendation. Results of a previous cost-effectiveness analysis assessing diet quality and costs for different breakfast menus also showed savings attributable to higher adherence in relation to the recommendation of fresh fruit consumption of the WHO Global Strategy [6]. Thus, strategies to encourage the consumption of fruits and vegetables may comprise suitable actions to provide high-quality diets at a low cost.

Similar, to our findings, the higher adherence to nutritional recommendations, measured by diet quality indices, has been associated with higher diet costs, especially among individuals from high-income countries, e.g., Belgium, the United States, Malaysia, and the United Kingdom [9,12,36,37,38,39].

A study based on data from the National Health and Nutrition Examination Survey (2007–2010) of the United States indicated a statistically significant positive association between diet quality and costs, measured by the Health Eating Index (HEI-2010) [38]. A similar result was identified in Malaysia, using nutritional recommendations from the Malaysian Healthy Eating Index (M-HEI) [39], and in the UK, using the dietary pattern proposed by the Dietary Approaches to Stop Hypertension (DASH) [9]. In Belgium, the diet cost was significantly higher for individuals with greater adherence to dietary recommendations from the country’s Supreme Health Council food guide, which follows WHO recommendations [12].

In Brazil, there is contradictory evidence regarding associations between diet quality and costs. Although certain studies showed higher cost associated with higher diet quality [7,8,13], other studies showed an absence of differences in cost [14] or lower costs associated with higher diet quality [6]. However, it is important to emphasize that our study showed potential causes of the divergences in cost-effectiveness ratios estimated in previous studies, i.e., adoption of diverse measures of diet quality (why), and effects of individuals’ personal and contextual characteristics, particularly referring to location of food purchases (where) and food choices (what) according to income and social class.

Regarding methodological issues (why), our results showed that the cost-effectiveness ratio of diets is highly dependent on the choice of nutritional recommendations that represent the health outcome within the economic assessment. The adoption of WHO recommendations on fruit, vegetables, sodium, sugar, and saturated fat intake showed lower costs with a higher diet quality than diets without adherence to the four recommendations.

Referring to the location of food purchases (where), there are substantial differences in variety and prices of food products according to the type of retail outlet in Brazil (street markets, supermarkets, wholesalers, whole foods markets, among others). In the present study, the accessibility to retail food outlets with a wide variety of fruits and vegetables, e.g., street markets, represented higher adherence to nutritional recommendations associated with lower cost; corroborating evidence from other studies conducted in Brazil [40,41]. In addition to high competition among suppliers, there is substantial decrease in prices from the beginning to the end of the fair to minimize losses due to the perishability of fresh foods, a phenomenon called “xepa” in Brazil. The “xepa” represents an additional opportunity to acquire fruits and vegetables at low prices, encouraging their consumption [42].

Regarding food choices (what) according to income and social class, the results of the study indicated that animal source foods, especially the meat group (protein food sources), presented high influence on the diet costs, regardless of nutritional quality. However, there were substantial differences in relation to the type of meats consumed according to the level of diet quality: individuals with higher diet quality selected animal source foods with lower sodium, added sugar, and saturated fat contents compared to individuals with lower diet quality. The results of the present study were consistent with previous evidence from the literature [12,39,43,44], pointing to high consumption of meats in relation to nutritional recommendations and higher participation of red meats and chicken in the cost of diets with higher and lower diet quality, respectively.

The relationship between income, social class, and diet costs identified in our study was consistent with other evidence in the literature [19,20]. In general, increasing socioeconomic level was associated with improvements in the nutritional quality of the diet. Individuals of higher social class (A) presented an increase of IM 0.83 per point of the BHEI-R in relation to individuals of lower social class (D + E), in addition to having a lower proportion of income committed by food expenses (17% in class A versus 55% in class D + E). A substantial proportion of household income was compromised by food expenditures among low-income families and individuals from lower social classes, representing a barrier for the improvement of the diet quality of individuals in vulnerable conditions.

The results were similar to evidence from another Brazilian study showing that households with lower income levels presented food expenditures corresponding to 90% of their income between 2008–2009 [7], showing the importance of conditional cash transfer programs to ensure food security among lower income individuals [45,46,47]. Recently published information from the Brazilian Household Budget Survey conducted in 2017–2018 pointed to food expenses being six times higher among richer families in comparison to poorer families at national level [45]. The evidence of the present study supports the implementation of public policy strategies based on subsidies applied to fresh foods and taxes applied to processed foods, which may reinforce other initiatives promoting healthy eating by changing relative prices without compromising the public sector budget [48].

It is important to consider the limitations of the present study. First, the ISA-Capital survey comprises cross-sectional data; thus, there is no possibility of establishing causal relationships in the context of the study. In addition, engagement of individuals in the survey may result in differences in cost-effectiveness analysis, given that individuals refusing to participate in the survey may represent another subset of the population group. However, statistical methods adopted for sample selection were designed to minimize eventual bias in the survey [28].

Second, data on prices adopted for estimation of diet costs were obtained from the Household Budget Survey from the Brazilian Institute for Geography and Statistics in 2008–2009; therefore, they may not correspond exactly to values of food items acquired by individuals interviewed in the ISA-Capital in 2015. However, we highlight that the use of robust linkage procedures ensured the estimation of reliable diet costs through the identification of similarities in geographical, period, and sociodemographic characteristics of households to allow matching between datasets [28].

Third, the absence of Household Budget Survey conducted in 2015 was solved through application of prices from the Household Budget Survey performed in 2008–2009. Nevertheless, it is important to emphasize that we adopted specific deflators calculated individually for each food item to update prices for 2015 to ensure the incorporation of changes in relative food prices of food items throughout the period, in addition to applying identical linkage procedures previously described to match households.

Finally, it is important to acknowledge the importance of evidence on cost-effectiveness of diets to inform public policy decision-making processes tackling food insecurity in low- and medium-income countries, particularly investigating the role of food prices, access, and affordability according to income level. Food insecurity increased during pandemics in various countries, especially in Brazil, making it vital to maintain and enhance government programs directed toward food security during uncertain periods.

## 5. Conclusions

This study identified higher diet costs associated with higher adherence to Brazilian dietary recommendations compared to diets with lower quality using a priori dietary patterns. However, the higher adherence to WHO recommendations represented lower diet costs. Animal source foods, particularly meats, represented the highest share in the diet costs, regardless of diet quality. However, vegetables and fruits and sweet and salty snacks represented a variable share of diet costs, depending on the quality of the diet of individuals living in Sao Paulo city. Furthermore, we identified lower diet costs among individuals with lower income and lower social class, associated with worse diet quality and a higher proportion of food expenditures in relation to the household income. In addition, the access to food retail with a wide variety and availability of fresh foods, e.g., street markets, represented an opportunity in the Brazilian food environment to improve the nutritional quality of the diet at a lower cost; therefore, we should create a continuous strategy in public policies toward food security in Brazil. The differences in the cost-effectiveness of diets were attributable to methodological choices in measuring dietary quality (why) and individuals’ contextual and personal characteristics, particularly, access to retail equipment (where) and food choices (what). Thus, cost-effectiveness analysis should be tailored to policy goals and local environments to ensure improvements in nutritional diet quality at lower cost.

## Figures and Tables

**Table 1:** Diet quality, cost, and cost-effectiveness ratio, according to individuals and household socioeconomic, demographic variables, and food purchase profile. ISA-2015, Sao Paulo, Brazil.

Characteristics	Total			BHEI-R			Cost (IM/Day)			CER		ICER ^a^	Income (IM/Capita/Day)	Diet Share in Income (%)
	*n*	%	CI 95%	μ	SE	*p*	μ	CI 95%	*p*	μ	SE			
Sex						0.394			**<0.001**					
Male	837	50.02	[47.17; 52.86]	58.12	0.50		9.71	[9.51; 9.91]		0.18	0.01		31.70	30.64
Female	905	49.98	[47.14; 52.83]	58.64	0.49		11.31	[11.09; 11.52]		0.20	0.01	3.08	33.25	34.01
Household income per capita ^b^						**<0.001**								
≤1 MW	769	50.07	[45.13; 55.00]	56.72	0.53		9.86	[9.67; 10.05]	**<0.001**	0.18	0.01		9.91	99.49
>1 MW	640	49.93	[45.00; 54.87]	59.35	0.55		11.23	[10.98; 11.48]		0.20	0.01	0.52	52.82	21.26
Socioeconomic level ^c^						0.256								
A	42	2.90	[1.91; 4.36]	61.07	2.62		11.96	[11.27; 12.66]	**<0.001**	0.22	0.02		71.13	16.82
B1 + B2	430	27.80	[24.57; 31.27]	58.85	0.73		11.25	[10.98; 11.52]		0.20	0.01	0.32	54.48	20.65
C1 + C2	968	53.60	[49.79; 57.36]	57.90	0.54		10.16	[9.95; 10.37]		0.19	0.01	0.57	23.47	43.28
D + E	304	15.71	[13.46; 18.26]	58.70	0.73		9.99	[9.70; 10.28]		0.18	0.01	0.83	18.17	55.02
Skin color						0.436								
White	611	51.24	[45.76; 53.41]	58.62	0.51		11.28	[11.05; 11.51]	**<0.001**	0.21	0.01		40.67	27.74
Other than white	569	48.76	[46.58; 54.24]	58.08	0.53		9.71	[9.52; 9.90]		0.17	0.01	2.91	24.45	39.71
Place of food purchase ^d^					0.516									
Minimarket	26	1.70	[0.70; 4.05]	54.78	-		8.21	-	**0.002**	0.18	-		13.56	60.58
Supermarket	1266	78.30	[69.27; 85.24]	58.56	0.41		9.64	-		0.19	0.01	0.38	33.33	28.92
Wholesaler	266	19.51	[12.70; 28.77]	57.74	1.14		10.70	[9.98; 11.42]		0.21	0.01	0.84	33.91	31.55
Whole foods market	10	0.49	[0.20; 11.78]	58.18	-		11.33	[9.98; 12.68]		0.15	-	0.92	21.41	52.92
Buy in street markets						**0.026**								
No	562	32.53	[29.22; 36.02]	57.92	0.64		11.27	[9.90; 12.64]	**0.030**	0.20	0.01		32.42	34.77
Once a month	98	6.17	[4.77; 7.95]	56.78	1.35		11.06	[9.88; 12.23]		0.20	0.01	0.18	28.29	39.08
Once–twice a month	225	14.70	[12.03; 17.83]	58.95	1.00		10.46	[9.46; 11.47]		0.19	0.01	-0.79	28.08	37.26
≥1 time a week	744	46.60	[42.24; 51.02]	59.07	0.54		9.91	[9.80; 11.99]		0.30	0.01	-2.04	61.58	30.10
Diet quality ^e^						**<0.001**			**<0.001**					
1st quintile	349	20.16	[17.77; 22.78]	40.23	0.43		10.25	[9.90; 10.60]		0.25	0.01		21.02	48.77
2nd quintile	348	20.89	[18.90; 23.03]	52.36	0.15		10.26	[9.99; 10.52]		0.19	0.01	0.00	28.40	36.10
3rd quintile	349	19.69	[17.48; 22.10]	59.56	0.11		10.34	[10.06; 10.62]		0.18	0.02	0.00	24.67	41.90
4th quintile	348	19.89	[17.85; 22.10]	65.78	0.11		10.58	[10.28; 10.88]		0.17	0.01	0.01	38.05	27.81
5th quintile	348	19.37	[17.32; 21.59]	74.96	0.29		11.12	[10.85; 11.39]		0.16	0.01	0.03	52.49	21.19

CER: Cost-effectiveness ratio; ICER: Incremental cost-effectiveness ratio; MW: Minimum wage; CI95%: 95% Confidence interval; μ: Mean; SE: Standard error; Analyses considered complex sample design. ^a^ Reference categories for ICER estimation: male sex, income ≤1 MS per capita, social class A, white skin color, usually buy at minimarkets, do not buy at open fairs, 1st. quintile of diet quality; ^b^ Minimum wage in 2015: IM 457.61. ^c^ According to the Brazil Economic Classification Criterion [2015] of the Brazilian Research Association (ABEP). ^d^ Main location for food purchases. ^e^ Estimated through the Revised Health Eating Index for the Brazilian population (BHE-R). **Bold** indicate statistical significance (*p* < 0.05).

**Table 2:** Diet cost in quintiles of nutritional quality, according to socioeconomic, demographic variables, and food purchase profile. ISA-2015, Sao Paulo, Brazil.

Characteristics	Low Adherence (Q1)			Q2			Medium Adherence (Q3)			Q4			High Adherence (Q5)			Nptrend ^d^
	μ	CI 95%	*p*	μ	CI 95%	*p*	μ	CI 95%	*p*	μ	CI 95%	*p*	μ	CI 95%	*p*	
Sex			**<0.001**			**<0.001**			**<0.001**			**<0.001**			**0.002**	
Male	9.52	[9.07; 9.98]		9.51	[9.20; 9.83]		9.46	[9.12; 9.80]		9.67	[9.34; 10.01]		10.55	[10.11; 10.98]		**<0.001**
Female	11.00	[10.53; 11.47]		10.97	[10.62; 11.32]		11.31	[11.03; 11.59]		11.55	[11.23; 11.88]		11.77	[11.38; 12.15]		**<0.001**
Household income per capita ^a^			**<0.001**			**<0.001**			**0.001**			**0.002**			**<0.001**	
≤1 MW	9.62	[9.29; 9.95]		9.51	[9.15; 9.87]		9.92	[9.54; 10.31]		10.05	[9.58; 10.52]		10.36	[10.01; 10.70]		**<0.001**
>1 MW	11.21	[10.64; 11.78]		11.12	[10.75; 11.48]		10.91	[10.49; 11.32]		11.15	[10.69; 11.61]		11.72	[11.36; 12.08]		**0.001**
Socioeconomic level ^b^			**<0.001**			**<0.001**			**0.003**			**0.005**			**<0.001**	
A	11.34	-		11.52	-		10.75	-		12.72	-		12.94	[11.58; 14.30]		**0.015**
B1 + B2	11.07	[10.48; 11.67]		11.31	[10.79; 11.83]		10.98	[10.55; 11.4]		11.23	[10.62; 11.84]		11.71	[11.23; 12.20]		**0.005**
C1 + C2	10.01	[9.63; 10.39]		9.83	[9.52; 10.14]		10.01	[9.65; 10.37]		10.19	[9.84; 10.55]		10.80	[10.45; 11.15]		**<0.001**
D + E	9.14	[8.50; 9.78]		9.79	-		10.05	[9.47; 10.62]		10.41	[9.58; 11.24]		10.53	[10.12; 10.95]		**<0.001**
Skin color			**<0.001**			**<0.001**			**<0.001**			**<0.001**			**<0.001**	
White	11.16	[10.66; 11.66]		11.03	[10.71; 11.34]		11.02	[10.70; 11.34]		11.53	[11.11; 11.95]		11.71	[11.4; 12.02]		**<0.001**
Other than white	9.52	[9.15; 9.89]		9.29	[8.95; 9.62]		9.55	[9.21; 9.88]		9.83	[9.49; 10.16]		10.41	[10.03; 10.80]		**<0.001**
Place of food purchase ^c^			0.059			0.116			**0.030**			0.345			0.957	
Minimarket	9.75	-		-	-		6.53	[−1.24; 14.29]		10.10	-		11.01	-		0.648
Supermarket	8.20	-		10.51	-		9.23	-		15.18	-		7.65	-		0.586
Wholesaler	9.90	[8.79; 11.01]		9.60	[8.97; 10.23]		10.97	[8.16; 13.77]		10.48	[9.37; 11.60]		12.57	[11.36; 13.77]		**<0.001**
Whole foods market	9.77	[7.86; 11.69]		10.64	-		11.53	-		12.77	[9.56; 15.98]		12.17	[10.14; 14.20]		0.085
Buy in street markets			0.352			0.877			0.287			0.522			0.273	
No	10.42	[8.59; 12.25]		9.99	[9.11; 10.87]		13.12	[6.20; 20.04]		11.30	[9.44; 13.15]		11.99	[10.54; 13.43]		**<0.001**
Once a month	9.30	[7.15; 11.46]		9.98	[8.32; 11.64]		10.13	-		11.38	[9.55; 13.21]		16.05	[10.09; 22.00]		**0.004**
Once–twice a month	9.09	-		9.73	[8.10; 11.37]		10.15	[7.65; 12.64]		11.04	[9.10; 12.98]		12.17	[9.31; 15.03]		**0.016**
≥1 time a week	9.51	[7.65; 10.65]		9.01	[8.04; 9.98]		9.76	[8.68; 10.85]		10.96	[9.19; 12.73]		11.20	[8.80; 12.92]		**<0.035**
Total	10.25	[9.89; 10.61]		10.26	[9.99; 10.52]		10.34	[10.06; 10.62]		10.58	[10.28; 10.88]		11.12	[10.85; 11.39]		**<0.001**

CER: Cost-effectiveness ratio; ICER: Incremental cost-effectiveness ratio; MW: Minimum wage; m: Mean; CI95%: 95% Confidence interval; Analyses considered complex sample design. ^a^ Minimum wage in 2015: IM 457.61. ^b^ Brazilian Economic Classification Criterion (2015) of the Brazilian Research Association (ABEP). ^c^ Main location for food purchases. ^d^ nptrend test, descriptive level *p* < 0.05. **Bold** indicate statistical significance (*p* < 0.05).

**Table 3:** Cost-effectiveness ratio, diet cost, and quality, according to WHO recommendations. ISA-2015, Sao Paulo, Brazil.

Recommendation	*n*	%	Cost			BHEI-R		CER		ICER ^a^
			μ	CI 95%	*p*	μ	SE	μ	SE	
Fruits, vegetables (400 g/day)										
Adherence	363	30.51	11.07	[10.81; 11.32]	**0.001**	70.73	0.79	0.21	0.03	
No adherence	825	69.49	11.76	[11.39; 12.14]		64.37	0.53	0.17	0.01	−0.11
Added sugar (<5% of total energy)					0.890					
Adherence	452	38.06	10.52	[10.17; 10.87]		58.90	0.44	0.20	0.01	
No adherence	736	61.94	10.49	[10.31; 10.68]		57.54	0.71	0.19	0.01	0.02
Saturated fat (<10% of total energy)					0.696					
Adherence	294	24.76	10.56	[10.2; 10.91]		58.50	0.46	0.19	0.01	
No adherence	894	75.24	10.49	[10.31; 10.67]		58.01	0.60	0.19	0.01	0.14
Sodium (<2000 mg/day)					0.735					
Adherence	346	29.09	10.55	[10.21; 10.88]		58.44	0.45	0.19	0.01	
No adherence	842	70.91	10.49	[10.3; 10.67]		58.24	0.74	0.19	0.01	0.3
WHO Recommendations										
Adherence to 4 criteria	54	4.52	10.97	[9.78; 11.41]	**0.010** ^b^	71.42	0.93	0.26	0.08	
Adherence at 3 criteria	150	12.60	11.17	[10.81; 11.54]		66.22	0.67	0.17	0.01	−0.04
Adherence at 2 criteria	377	31.77	11.20	[10.85; 11.56]		66.02	1.00	0.19	0.01	−0.04
Adherence to 1 criterion	455	38.33	11.25	[10.71; 11.78]		62.81	1.45	0.19	0.02	−0.03
No adherence	152	12.78	12.03	[11.38; 12.68]		64.49	-	0.15	-	−0.15

μ: mean; SE: Standard error; CI 95%: 95% Confidence interval; CER: Cost-effectiveness ratio; ICER: Incremental cost-effectiveness ratio; Analyses considered complex sample design. ^a^ Reference categories for ICER estimation: no adherence to the recommendations for consumption of fruits, vegetables, added sugar, saturated fat, and sodium and adherence to all four recommendations. ^b^ nptrend test, descriptive level *p* < 0.05. **Bold** indicate statistical significance (*p* < 0.05).

**Table 4:** Proportion of consumers, daily cost, and main contributing group, according to WWEIA classification and diet quality, adapted for Latin America. ISA-2015, Sao Paulo, Brazil.

Group	*n*	% ^a^	Cost ^b^	SE	% ^c^	*p* ^d^	Main Contributors	%	Cost
Low-quality diet ^e^									
Milk and dairy products	384	66.21	0.82	0.04	9.23	0.165	Whole milk	3.32	0.30
Proteins	488	84.14	2.18	0.13	24.38	<0.001	Meat, excluding ground meat	10.9	0.97
Mixed dishes	294	50.69	1.01	0.07	11.33	0.030	Rice-based dishes	2.96	0.26
Cereals/grains	547	94.31	0.70	0.03	7.88	0.016	Breads	4.23	0.38
Salty and sweet snacks	386	66.55	1.15	0.06	12.86	<0.001	Sweets with chocolate	2.68	0.24
Fruit	101	17.41	0.47	0.04	5.24	<0.001	Fruit salad and other fruits	1.75	0.16
Vegetables	307	52.93	0.98	0.04	10.98	0.997	French fries	2.73	0.24
Nonalcoholic beverages	545	93.97	1.44	0.05	16.12	<0.001	Coffee	4.83	0.43
Oils and fats	364	62.76	0.17	0.01	1.98	<0.001	Butter and animal fat	0.68	0.06
Intermediate diet quality ^e^									
Milk and dairy products	421	72.46	0.85	0.03	9.46	0.165	Whole milk	2.97	0.01
Proteins	551	94.84	2.66	0.09	29.47	<0.001	Meat, excluding ground meat	12.62	0.02
Mixed dishes	231	39.76	0.92	0.08	10.21	0.03	Rice-based dishes	2.10	0.01
Cereals/grains	575	98.97	0.8	0.03	8.88	0.016	Breads	4.45	0.01
Salty and sweet snacks	327	56.28	0.85	0.06	9.45	<0.001	Sweet cakes and pies	2.05	0.01
Fruit	257	44.23	0.6	0.03	6.68	<0.001	Banana	2.10	0.01
Vegetables	404	69.54	0.96	0.05	10.61	0.997	Potatoes	2.40	0.01
Nonalcoholic beverages	551	94.84	1.23	0.05	13.65	<0.001	Artificial fruit juices	4.71	0.01
Oils and fats	402	69.19	0.15	0.01	1.59	<0.001	Margarine	0.59	0.00
High diet quality ^e^									
Milk and dairy products	452	77.8	0.79	0.03	9.09	0.165	Skim milk	3.64	0.31
Proteins	565	97.25	2.56	0.11	29.56	<0.001	Chicken, whole pieces	11.05	0.96
Mixed dishes	206	35.46	0.93	0.12	10.7	0.030	Soups	2.98	0.26
Cereals/grains	568	97.76	0.77	0.03	8.94	0.016	Breads	4.13	0.36
Salty and sweet snacks	254	43.72	0.57	0.04	6.66	<0.001	Cakes and pies	1.48	0.13
Fruit	443	76.25	0.68	0.02	7.85	<0.001	Banana	2.62	0.23
Vegetables	453	77.97	1.12	0.08	12.93	0.997	Potatoes	3.38	0.29
Nonalcoholic beverages	540	92.94	1.12	0.04	12.93	<0.001	Coffee	4.69	0.41
Oils and fats	348	59.90	0.12	0.01	1.35	<0.001	Salad dressings and vegetable oils	0.57	0.05

SE: Standard error; Analyses considered complex sample design. ^a^ Percentage of consumers. ^b^ In International Monetary units (IM); adjusted for sex, age, per capita family income, social class, diet quality, place of purchase, energy, and grams. ^c^ Percentage of food group contribution in relation to diet cost. ^d^ nptrend test, descriptive level *p* < 0.05. ^e^ Estimated through the Revised Health Eating Index for the Brazilian population (BHEI-R). **Bold** indicate statistical significance (*p* < 0.05).

## Data Availability

The datasets generated during and/or analysed during the current study are available from the corresponding author on reasonable request.
